# Systematic Screening of Communication Outcomes at Age 5 in Children Treated for Nonsyndromic Craniosynostosis

**DOI:** 10.1111/1460-6984.70305

**Published:** 2026-08-03

**Authors:** Justin Weinfeld, Christina Havstam, Marizela Kljajić, Clin Psychol, Lars Kölby, Peter Tarnow, Christina Persson

**Affiliations:** ^1^ Institute of Neuroscience and Physiology, Department of Health and Rehabilitation, Speech and Language Pathology Unit, Sahlgrenska Academy University of Gothenburg Gothenburg Sweden; ^2^ Department of Otorhinolaryngology Sahlgrenska University Hospital Gothenburg, Region Västra Götaland Sweden; ^3^ Department of Plastic Surgery Sahlgrenska University Hospital Gothenburg, Region Västra Götaland Sweden; ^4^ Institute of Clinical Sciences, Department of Plastic Surgery, Sahlgrenska Academy University of Gothenburg Gothenburg Sweden

**Keywords:** communication, non‐syndromic craniosynostosis, screening

## Abstract

**Background:**

Previous research on communicative outcomes in children treated for nonsyndromic craniosynostosis (NSC) has yielded inconsistent findings, with reported prevalence rates of communicative difficulties varying widely. These discrepancies are attributable to small samples, heterogeneous age groups, and reliance on indirect or non‐domain‐specific outcome measures. There remains a need for systematic, age‐specific screening using validated tools to clarify the nature and prevalence of communicative vulnerabilities in this population.

**Aims:**

The primary aim was to assess guardian‐reported communicative abilities in a consecutive cohort of 5‐year‐old children treated for NSC and compare outcomes with normative data. Secondary aims were to examine whether reported difficulties were predominantly speech‐language based or pragmatic, and to explore differences related to NSC subtype and timing of surgery.

**Methods and Procedures:**

This cross‐sectional study included 157 children treated for NSC at a national craniofacial centre in Sweden. Guardians completed the Children's Communication Checklist–2 (CCC‐2) as part of routine 5‐year follow‐up. General Communication Composite (GCC) scores and subscale profiles were analysed and compared with published normative data. Group differences across synostosis type and surgical timing were examined using parametric and non‐parametric statistical methods.

**Outcomes and Results:**

Eighty‐five percent of children scored within the normative range on the GCC. Fifteen percent scored below the clinical cut‐off, a proportion not significantly different from the normative sample. Group‐level differences were primarily observed in the domains of speech and language, with significantly lower scores in speech and syntax, while pragmatic difficulties were uncommon. No significant differences were found between craniosynostosis subtypes. Earlier surgery was associated with higher GCC scores in children with metopic NSC only.

**Conclusion and Implications:**

Most children treated for NSC demonstrate age‐appropriate communicative abilities at 5 years of age. However, subtle vulnerabilities in speech and syntax were present in a subset of children. These findings support the use of screening in routine follow‐up to identify children who may benefit from further assessment, while suggesting that broad language disorder–like profiles are uncommon in this population.

**WHAT THIS PAPER ADDS:**

*What is already known on this subject*

- Previous studies report highly variable rates of speech and language difficulties in children treated for nonsyndromic craniosynostosis (NSC), ranging from low to markedly elevated prevalence.

*What this study adds to the existing knowledge*

- In a large consecutive cohort of 5‐year‐old children treated for NSC, 85% showed age‐appropriate communicative abilities as rated by their guardians and did not differ significantly from normative data. Group‐level differences were confined to speech‐ and language domains (speech and syntax), while pragmatic difficulties were uncommon. Earlier surgery was associated with higher communication scores in children with metopic NSC only.

*What are the potential or actual clinical implications of this study?*

- Routine screening at follow‐up is supported to identify speech‐ and language vulnerabilities in a minority of children treated for NSC. Broad language disorder–like profiles appear uncommon, suggesting that monitoring of speech and syntax may be more clinically relevant than global language screening alone.

## Key Points

Most 5‐year‐old children treated for nonsyndromic craniosynostosis had guardian‐reported communication abilities within the normative range, and the proportion scoring below the CCC‐2 clinical cut‐off did not differ significantly from normative data.

Communication vulnerabilities were mainly observed in speech and syntax, whereas pragmatic difficulties and broad language disorder–like profiles were uncommon.

Systematic communication screening can support routine craniofacial follow‐up by identifying children who require comprehensive speech and language assessment, without subjecting all children to extensive assessment solely on the basis of their craniosynostosis diagnosis.

## 1 Introduction

Language ability encompasses multiple domains including speech, receptive and expressive language, and pragmatics that together shape a child's functional communication. When one or more of these domains are compromised, the risk of persistent difficulties in learning, social interaction, and daily functioning increases (Norbury et al. 2016). Despite the importance of these skills, existing research on communicative outcomes in children with nonsyndromic craniosynostosis (NSC) shows wide variability, with reported prevalence rates of speech‐ and language difficulties differing substantially across studies. This inconsistency reflects several methodological limitations in the current literature, including small sample sizes (Mendonca et al. 2009; Virtanen et al. 1999), reliance on broad neuropsychological measures rather than domain‐specific communication tools (Magge et al. 2002), frequent use of indirect proxies such as therapy referral (Becker et al. 2005; Naran et al. 2017; Tandon et al. 2021), or no information on assessment tools used (Mendonca et al. 2009; Tandon et al. 2021). These gaps underscore the need for systematically collected data using validated, domain‐specific screening measures to clarify the nature and prevalence of communicative vulnerabilities in children treated for NSC and to better inform clinical follow‐up practices.

The term NSC is commonly used to describe individuals with craniosynostosis in whom no additional congenital anomalies or known genetic syndromes have been identified. However, this terminology is inherently problematic, as “nonsyndromic” does not imply the absence of underlying genetic or neurodevelopmental influences (Posey et al. 2017). Despite this limitation, the term remains widely used in both clinical practice and research to denote individuals treated for craniosynostosis without an identified associated syndrome, and it is therefore retained in the present study for consistency with existing literature.

NSC, characterized by the premature fusion of cranial sutures, has been linked to a range of cognitive, speech, language, behavioural and psychiatric differences (Aksoğan et al. 2024; Becker et al. 2005; Chieffo et al. 2010; Kelleher et al. 2006; Korpilahti et al. 2012; Magge et al. 2002; Naran et al. 2017; Harrison et al. 2026; Shipster et al. 2003; Starr et al. 2012; Tandon et al. 2021; Tillman et al. 2020; Virtanen et al. 1999). Speech‐language difficulties in this population varies notably; from 2.3% to 56.4% (Naran et al. 2017; Stanbouly et al. 2024), and is commonly reported as more common than in the general paediatric population, which is estimated at a rate of 7%–8% (Norbury et al. 2016) and additionally with a significant number of children requiring speech therapy (Becker et al. 2005; Kelleher et al. 2006; Naran et al. 2017; Shipster et al. 2003; Tandon et al. 2021). However, while NSC in previous research is associated with speech‐language difficulties, the overall cognitive ability of children with this condition have been reported to be within the average range, suggesting that the speech‐ language difficulties are specific rather than indicative of a general cognitive delay (Da Costa et al. 2006; Kljajić et al. 2019; Shipster et al. 2003; Speltz et al. 2015). In summary, studies differ significantly in reported prevalence of speech‐ language difficulties in this clinical group (Aksoğan et al. 2024; Kelleher et al. 2006; Korpilahti et al. 2012; Naran et al. 2017; Shipster et al. 2003; Starr et al. 2012).

Some studies have identified differences in neurodevelopment between types of NSC, indicating that individuals with sagittal NSC exhibit the lowest incidence of neurodevelopmental difficulties, including speech‐ and language difficulties (Junn et al. 2023; Kalmar et al. 2022; Millichap 2015; Speltz et al. 2015; Starr et al. 2012). Contradictory findings regarding the impact of timing of surgery and surgical technique on cognitive ability have emerged in research (Chowdhury et al. 2023). Some studies have found that surgical correction performed before 6 months of age, and choice of surgical technique has been indicated to have a positive impact on speech‐ and language development in NSC (Alperovich et al. 2021; Lynn et al. 2023; Patel et al. 2014).

Given the high prevalence of speech‐ and language difficulties and their potential impact on long‐term outcomes in children treated for NSC, early and systematic screening for language difficulties at routine visits could inform clinicians if further assessments are needed, potentially mitigating difficulties in communicative domains. Comprehensive speech and language assessment by a speech‐language pathologist provides the most detailed information about a child's communicative abilities. However, in multidisciplinary team follow‐up routines, full assessment of all children is not always feasible. Many families travel long distances to attend the multidisciplinary visits. These visits involve several visits with clinicians within a limited time frame, and young children may not always be able to cooperate with lengthy standardised testing. Repeated assessment sessions may be impractical for families who live far from the centre. In this context, systematic screening serves as a first step to identify children who may require more detailed speech‐ and language evaluation.

Screening for both speech‐ language and pragmatic communicative difficulties is relevant, as in addition to speech‐ and language difficulties, increased cognitive and behavioural differences are also reported in this clinical population (Almeida et al. 2024; Shimoji et al. 2015). Kilcoyne et al. (2021) emphasised that parental concern alone is insufficient to identify language difficulties in children with craniosynostosis, and advocated for systematic use of parent rated screening protocols. Their findings support the inclusion of structured tools such as the Children's Communication Checklist 2 in routine follow‐up, ensuring that subtle but clinically relevant vulnerabilities are not overlooked. The CCC‐2 (Bishop 2003) is a questionnaire intended for completion by guardians or individuals closely acquainted with the child being assessed. Its purpose is to screen for a broad range of communicative competencies in children aged 4–16; both speech‐ and language based (for example affecting speech, semantics or syntax) or pragmatic difficulties (e.g., affecting communicative initiative, stereotypical language or use of context). Furthermore, the CCC‐2 can be utilized to screen for aspects of other neurodevelopmental difficulties. Its primary function is to screen for such difficulties, enabling clinicians to determine if further assessments are warranted. Current evidence provides an incomplete and inconsistent picture of speech and language outcomes in NSC, particularly at specific developmental time points. The absence of large, age‐uniform cohorts constitutes a knowledge gap, which the present study addresses by examining communicative outcomes in a large, consecutive cohort of 5‐year‐old children treated for NSC.

The main aim of this study was to assess the communicative abilities reported by guardians in a cohort of 5‐year‐old children treated for NSC using a systematic screening approach and compare these results against normative data. Secondary objectives included investigating the nature of the communicative difficulties and exploring potential differences in scores on the outcome measure based on the craniosynostosis type and timing of surgery.

## 2 Materials and Method

### 2.1 Procedure

At the Craniofacial Centre at Sahlgrenska University Hospital, Gothenburg, Sweden, children treated for NSC undergo routine medical examination by a plastic surgeon and screening of speech and language ability by a speech‐language pathologist (SLP) at Ages 3 and 5. The centre is 1 of 2 in Sweden that perform treatment of craniosynostosis, and treats approximately 75% of cases in the country. At the Craniofacial Centre spring‐assisted osteotomy and distraction osteogenesis is used as primary surgical techniques, with cranial vault reconstruction as the technique of choice if the child is treated after 6 months of age (Kölby et al. 2017). In anticipation of their scheduled craniofacial team visit at 5 years of age, families receive the Children's Communication Checklist (CCC‐2) as part of a standardised screening procedure. Families are presented with the option to either submit the completed CCC‐2 prior to their appointment or bring it with them to the clinic. In addition to the CCC‐2, at the clinic children undergo an assessment of speech via a standardised instrument.

### 2.2 Participants

**FIGURE 1:**
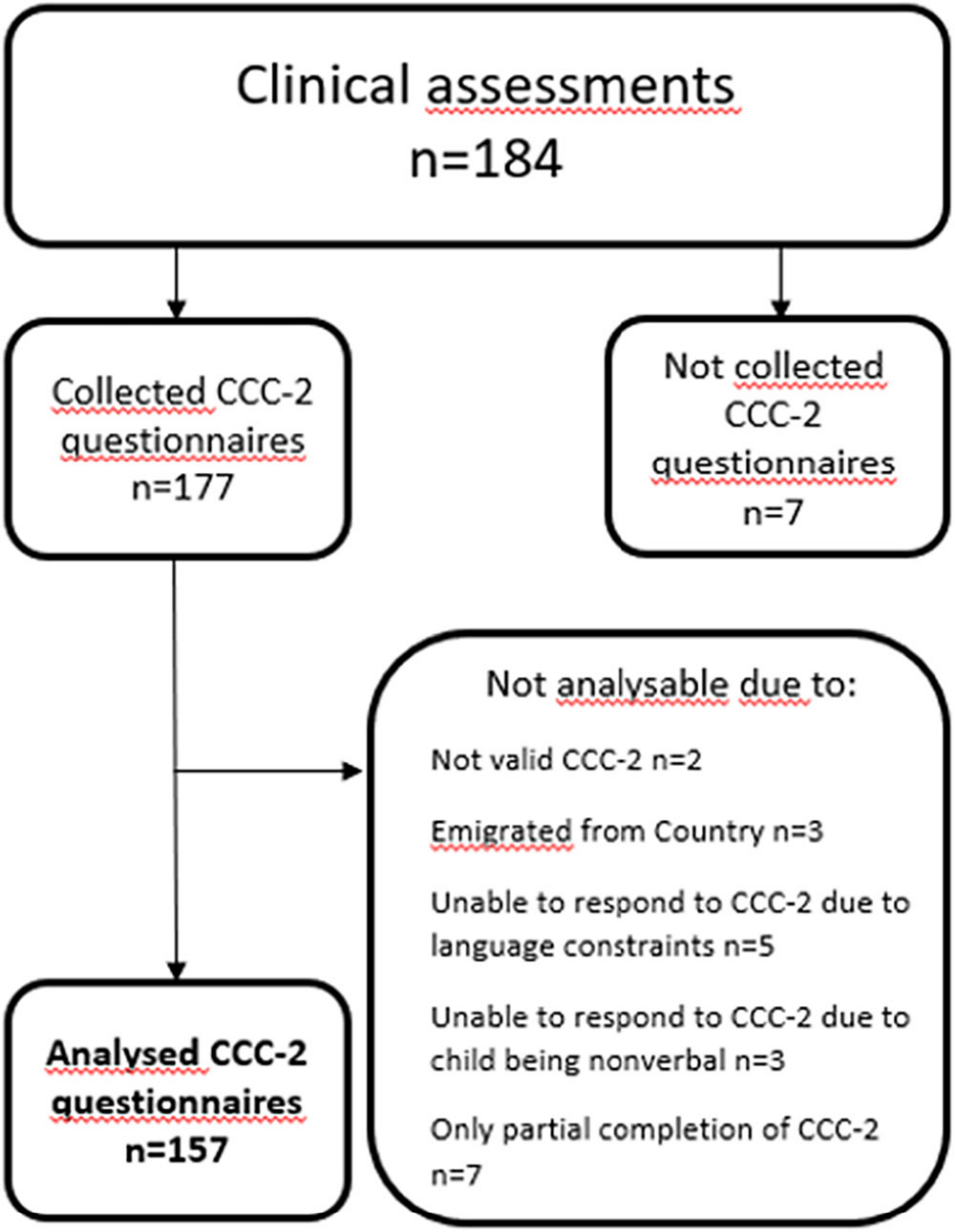
Flow chart of inclusion.

In a consecutive series of 184 children treated for NSC seen by the multidisciplinary craniofacial team at the clinic or via Telemedicine solutions between October 2020 and January 2025, 157 children's CCC‐2 were analysed (Figure 1). No exclusions were made on the basis of co‐occurring speech‐language difficulties, autism, ADHD, developmental delay, or other neurodevelopmental or behavioural diagnoses. This was intentional, as the aim was to describe communication outcomes in the clinical population encountered in routine craniofacial follow‐up rather than to estimate outcomes in a highly selected subgroup without additional developmental vulnerabilities. Information on language background was obtained from guardian report and included the child's strongest language and whether more than one language was spoken in the home. Children were not excluded on the basis of multilingual exposure, provided that the CCC‐2 could be completed by a guardian familiar with the child's communication. Demographic data are presented in Table 1 and the participants have been grouped according to NSC‐type. In addition to the Sagittal, Metopic and Unicoronal NSC‐groups, a combined category labelled Miscellaneous NSC (MNSC) was created for statistical purposes to enable group‐level comparisons. This group includes participants with rare suture involvement: lambdoid (*n* = 4), multiple sutures (*n* = 4), bicoronal (*n* = 3), and frontosphenoidal (*n* = 1) NSC.

**TABLE 1:** Demographic and clinical characteristics by craniosynostosis type.

Variable	Sagittal	Metopic	Unicoronal	Miscellaneous	Overall
Sex, *n* (%)					
Male	59 (72)	32 (68)	6 (38)	5 (42)	102 (65)
Female	23 (28)	15 (32)	10 (63)	7 (58)	55 (35)
Age, months (IQR)	63 (3)	63 (3)	62 (3)	63 (3)	63 (3)
Age, months, min–max	58–70	59–74	59–68	59–67	58–74
CCC‐2 completed by, *n* (%)					
Mother	47 (57)	25 (53)	9 (56)	6 (50)	87 (55)
Father	14 (17)	12 (26)	4 (25)	2 (17)	32 (20)
Both	15 (18)	6 (13)	0 (0)	1 (8.3)	22 (14)
Other	0 (0)	0 (0)	0 (0)	1 (8.3)	1 (0.6)
Unknown	6 (7.3)	4 (8.5)	3 (19)	2 (17)	15 (9.6)
Language spoken at home, *n* (%)					
Swedish	76 (93)	40 (85)	15 (94)	10 (83)	141 (90)
Other	6 (7)	7 (15)	1 (6)	2 (17)	16 (10)
More than one language	10 (12)	11 (23)	3 (19)	2 (17)	26 (17)
Timing of surgery, *n* (%)					
<6 months	54 (66)	28 (60)	4 (25)	5 (42)	91 (58)
≥ 6 months	28 (34)	19 (40)	12 (75)	7 (58)	66 (42)
GCC, (SD)	78.7 (21.3)	75.3 (22.7)	74.8 (15.7)	72.2 (12.2)	76.8 (20.7)

*Note*: Miscellaneous NSC: lambdoid (*n* = 4), multiple sutures (*n* = 4), bicoronal (*n* = 3), frontosphenoidal (*n* = 1).

Abbreviations: CCC‐2, children's communication checklist; GCC, general communication composite; IQR, interquartile range.

### 2.3 Ethics

The study was conducted according to the principles stated in the Declaration of Helsinki, and was approved by the Swedish Ethical Review Authority. We employed an opt‐out consent process. All potential participants received a written information letter detailing the study aims, procedures, risks, and data management. Participation was considered agreed unless the individual contacted the research team to decline. If families opted out, they were excluded from all study procedures.

### 2.4 Instrument

The CCC‐2 (Bishop 2003) consists of 70 items, grouped into 10 subscales, each containing seven items; the first four subscales assess aspects of speech‐ and language (Speech, Syntax, Semantics and Coherence); the next four subscales focus on pragmatic language and social communication and two additional subscales assess social interaction and restricted interests. The Swedish manual reports internal consistency estimated for the CCC‐2 scales, with Cronbach's alpha values ranging from 0.69 to 0.88, supporting the reliability of the scale scores for screening purposes. Each item is rated on a 4‐point Likert scale according to how often the described behaviour occurs. Raw scores for each subscale are converted to scaled scores based on normative data. A General Communication Composite (GCC) score is derived by summing the scaled scores from the first eight subscales. The GCC provides an overall index of communicative competence, with lower scores indicating greater difficulties. The CCC‐2 provides a defined cut‐off point (≤54 points) to indicate clinically significant communication difficulties. Scores below this threshold suggest that the child's overall communicative competence is substantially lower than expected for age and indicates a recommendation for further evaluation. The CCC‐2 has been translated into Swedish, with norms available for Swedish‐speaking children (Bishop 2011).

### 2.5 Statistics

Statistical analyses were conducted using R version 4.4.2 (R core team 2024) and IBM SPSS Statistics version 28.0.1.1 (IBM corp., Armonk NY). Descriptive statistics were computed for all variables. Group differences in categorical outcomes were examined using chi‐square tests, with Fisher's exact test applied when expected cell counts were small. To compare mean GCC scores across the four craniosynostosis subtypes, a one‐way ANOVA was performed. Welch's two‐sample *t*‐tests, calculated from published summary statistics for the normative sample, were used for group comparisons. Effect sizes were reported as Cramer's V for categorical comparisons and Hedges’ g for mean differences. A MANOVA was conducted to assess differences across craniosynostosis subgroups on the ten CCC‐2 subscales, and follow‐up univariate ANOVAs were performed when applicable. To examine whether surgical timing had any impact on GCC a linear model with an interaction between timing of surgery and synostosis type was used. Statistical significance was defined as p < .05 for all analyses.

## 3 Results

### 3.1 Attrition Analysis

Attrition analysis was conducted comparing children whose data were analysed in the study with those whose data were not, excluding data from three families that had emigrated from the country and could not be contacted. All variables were non‐significant except the distribution of sex (Table 2). No families contacted opted out from participation.

**TABLE 2:** Attrition analysis.

Characteristics	Loss *N* = 24	Participants *N* = 157	*p* value
Age, months^a^	63 (61, 64)	63 (61, 64)	0.8
Sex, *n* (%)			0.028
Male	3	55	
Female	21	102	
NSC type, *n* (%)			0.8
Sagittal	11	82	
Metopic	7	47	
Unicoronal	3	16	
Miscellaneous	3	16	
Timing of Surgery, *n*			0.2
<6 months	17	91	
≥6 months	7	66	

^a^ Median (Q1, Q3).

### 3.2 General Communication Composite

**FIGURE 2:**
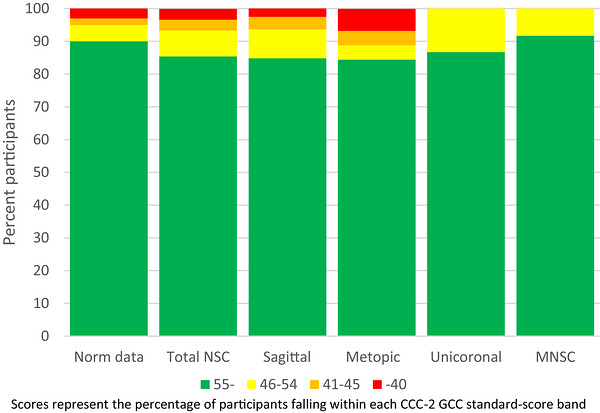
CCC‐2 GCC scores compared to norm data. CCC‐2 GCC standard‐score bands correspond approximately to the following percentiles: ≥55 = ≥10th percentile; 46–54 = 5th–10th percentile; 41–45 = 3rd–5th percentile; ≤40 = <3rd percentile.

Analysis of the General Communication Composite (GCC), revealed that 85% of children across all synostosis groups reported no speech‐ or language difficulties as assessed by their guardians (Figure 2). For each NSC group the proportion of children scoring above the cut‐off indicating age‐appropriate language skills was over 80%. Of the children scoring below the cut‐off indicating language difficulties, 88% (*n* = 21) showed a profile of speech‐language difficulties, and 12% (*n* = 3) a profile of language‐pragmatic difficulties.

In the NSC group, 15% (24 out of 157; 95% CI [10.5%, 21.7%]) of participants scored below the GCC clinical cutoff (≤54) on the CCC‐2, compared to 10% (46 out of 451; 95% CI [7.7%, 13.3%]) in the norm group. The miscellaneous NSC group had the lowest proportion of children scoring below the cut‐off (8%), followed by the sagittal (15%), metopic (17%) and unicoronal (19%) groups. Further analysis of the proportion of children in the NSC group scoring below the 10th percentile (GCC score 46–54), below the 5th percentile (GCC score 41–45) and below the 3rd percentile (GCC score ≤40) for each NSC type were performed to examine whether the distribution of CCC‐2 scores differed across the four NSC types and the norm data. The overall comparison across the normative sample, the total NSC group, and the different craniosynostosis subtypes (sagittal, metopic, unicoronal, and MNSC) was not significant, *χ* ^2^(15) = 15.03, *p* = 0.45. Pairwise comparisons between the normative sample and each craniosynostosis group also showed no significant differences: Total NSC, *χ* ^2^(3) = 3.94, *p* = 0.27; sagittal, *χ* ^2^(3) = 2.58, *p* = 0.46; metopic, *χ* ^2^(3) = 5.05, *p* = 0.17; unicoronal, *χ* ^2^(3) = 6.13, *p* =  011; and MNSC, *χ* ^2^(3) = 0.85, *p* = 0.84. The results indicate that the distribution of GCC scores does not differ systematically between the normative population, the overall NSC group, or the NSC craniosynostosis types. To compare the four NSC‐types on the mean GCC‐score, one‐way ANOVA analysis was performed and did not reveal any statistical differences between the groups, F(0.30) = 380, p. 83.

### 3.3 The Speech and Syntax Subscales of the CCC‐2

Two‐sample *t*‐tests revealed significant differences between the normative sample and the craniosynostosis group on the *Speech* (measures intelligibility and fluency in speech) subscale, t(210.59) = 4.13, *p* < 0.001, and the Syntax (measures sentence structure and grammar) subscale, *t*(209.93) = 4.64, *p* < 0.001.The study group scored lower in both domains. Mean differences were 1.58 (95% CI [0.83, 2.33]) for Speech and 1.57 (95% CI [0.91, 2.23]) for Syntax, with moderate effect sizes (*g* = 0.45 and 0.51, respectively), see Table 3. Independent samples t‐tests were conducted to compare CCC‐2 subscale scores between the norm group (*n* = 451) and four craniosynostosis subgroups: sagittal (*n* = 82), metopic (*n* = 47), unicoronal (*n* = 16), and miscellaneous (*n* = 12) (Table 4). Overall, *Syntax* consistently showed group differences across all comparisons, and *Speech* showed moderate to large effects in two groups, see Table 4. A MANOVA was conducted to compare CCC‐2 subscale scores across the four craniosynostosis groups. The test was not significant using Wilks’ Lambda, Λ = 0.776, F(30, 423.34) = 1.27, *p* = 0.158, partial *η* ^2^ = 0.081, follow‐up univariate ANOVAs showed no significant group differences on individual subscales (all *p* > 0.05).

**TABLE 3:** CCC‐2 subscale scores in children with nonsyndromic craniosynostosis compared with published normative data.

Subscale	Mean (NSC	Mean (Norm)	Mean diff (Norm‐NSC)	Hedge's *g*	*t*	df	*p*	*p* (Holm)
A. Speech	8.62	10.20	1.58	0.45	4.13	210.59	<0.001***	<0.001***
B. Syntax	8.58	10.15	1.57	0.51	4.64	209.93	<0.001***	<0.001***
C. Semantics	9.65	9.96	0.31	0.10	1.09	290.89	0.275	1.000
D. Coherence	9.57	10.14	0.57	0.17	1.65	239.12	0.100	0.803
E. Initiative	10.17	10.32	0.15	0.05	0.54	270.50	0.587	1.000
F. Stereotypical language	9.73	10.06	0.33	0.12	1.21	234.70	0.229	1.000
G. Use of context	10.39	10.33	−0.06	−0.02	−0.20	367.72	0.841	1.000
H. Nonverbal communication	9.75	10.07	0.32	0.11	1.13	255.16	0.259	1.000
I. Social relations	9.83	10.17	0.34	0.11	1.04	234.34	0.299	1.000
J. Interests	10.75	10.32	−0.43	−0.14	−1.53	264.45	0.127	0.892

*Notes*: Norm data derived from published data in the Swedish Manual of the CCC‐2 (2012).

Welch's *t*‐tests; Hedge's *g*; Holm‐adjusted *p* values; NSC *n* = 157, Norm *n* = 451.

**TABLE 4:** Speech and Syntax CCC‐2 Subscale Scores by Nonsyndromic Craniosynostosis Type Compared With Published Normative Data.

A. Speech									
	*n*	Mean	Mean (Norm)	Mean diff (Norm‐NSC type)	Hedge´s *g*	*t*	df	*p*	*p* (Holm)
Sagittal	82	8.95	10.20	1.25	0.38	2.45	95.73	0.016	0.048*
Metopic	47	8.36	10.20	1.84	0.57	2.79	50.78	0.007	0.030*
Unicoronal	16	7.19	10.20	3.01	0.95	2.35	15.39	0.033	0.066
MNSC	12	9.33	10.20	0.87	0.28	0.81	11.41	0.434	0.434

B. Syntax									
	*n*	Mean	Mean (Norm)	Mean diff (Norm‐NSC‐type)	Hedge's *g*	*t*	df	*p*	*p* (Holm)
Sagittal	82	9.04	10.15	1.11	0.38	2.47	95.71	0.015	0.030*
Metopic	47	8.32	10.15	1.83	0.63	2.80	49.72	0.007	0.022*
Unicoronal	16	8.00	10.15	2.15	0.79	2.52	15.70	0.023	0.030*
MNSC	12	7.25	10.15	2.90	1.07	3.87	11.67	0.002	0.009**

*Note*: Welch's *t*‐tests; Hedge's *g*; Holm‐adjusted *p* values, Norm *n* = 451.

Analysis of individual CCC‐2 items showed that the difficulties observed were largely consistent across NSC subtypes. Among children with the lowest scores (≤10th percentile), errors in articulation (such as mispronouncing “s” or “r”) and omission of grammatical markers (e.g., past tense endings) were the most frequent indicators.

### 3.4 Surgical Timing

Differences in General Communication Competence (GCC) between early (<6 months) and late (≥6 months) surgery were examined separately within each NSC type. Among children with sagittal NSC, mean GCC scores were similar between early and late surgery groups (early: 79.9 ± 21.3; late: 76.3 ± 21.6), with no statistically significant difference. In contrast, metopic NSC children who underwent early surgery had substantially higher GCC scores compared with those operated later (early: 80.9 ± 20.7; late: 67.1 ± 23.5), corresponding to a mean difference of 13.8 points (95% CI 1.8–25.9, *p* = 0.025). For unicoronal synostosis and MNSC, mean GCC scores differed in direction between early and late surgery groups; however, these comparisons were based on small sample sizes and were not statistically significant (Table 5)

**TABLE 5:** General communication composite (GCC) by timing of surgery (early vs. late surgery).

General communication composite by timing of surgery							
CCC‐2 GCC scores stratified by NSC type							
	Early surgery (<6 months): *n*	Late surgery (≥6 months): *n*	Early surgery (<6 months): Mean (SD)	Late surgery (≥6 months): Mean (SD)	Mean difference (Early–Late)	95% Confidence interval	*p* value
Sagittal NSC	54	28	79.9 (21.3)	76.3 (21.6)	3.60	[−5.86, 13.07]	0.453
Metopic NSC	28	19	80.9 (20.7)	67.1 (23.5)	13.84	[1.76, 25.92]	0.025^*^
Unicoronal NSC	4	12	81.8 (14.6)	72.4 (16.0)	9.33	[−14.14, 32.80]	0.433
MNSC	5	7	68.2 (7.4)	75.0 (14.6)	−6.80	[−6.80, 17.00]	0.573

*Note*: Positive mean differences indicate higher GCC scores in the early surgery group.

Abbreviations: CCC‐2, children's communication checklist; GCC, general communication composite.

* *p* < 0.05.

## 4 Discussion

The present findings suggest that speech‐ and language outcomes in children with NSC involve relatively subtle vulnerabilities. Most children in the cohort scored within the normative range on the CCC‐2, and the proportion scoring below the clinical cut‐off was not significantly different from the normative comparison group. Importantly, few children showed CCC‐2 profiles suggestive of language–pragmatic difficulties. Instead, group‐level differences were largely confined to the speech‐ language domains of syntax and speech. However, individual children do have more severe speech‐ and language issues, both as observed in the study group and evident in the fact that three children were excluded due to lack of speech.

The present findings can be discussed within a broader theoretical framework linking NSC to specific neurodevelopmental vulnerabilities (Ijichi et al. 2025; Kapp‐Simon et al. 2007; Speltz et al. 2004). Historically, differences in speech or language outcomes in NSC were explained as the downstream effects of restricted skull growth and elevated intracranial pressure (ICP) (Kalmar et al. 2024; Thiele‐Nygaard et al. 2020). However, more recent research suggests that the relationship between skull and brain is interactive: premature suture fusion alters the shape and organization of the developing brain in ways not limited to the region beneath the fused suture (Russo et al. 2024). Neuroimaging studies demonstrate cortical and subcortical dysmorphologies, including alterations in frontal lobes, corpus callosum, and perisylvian regions that are critical for language processing (Palaios et al. 2024). Even after corrective surgery, such subtle differences in neural organization may persist and contribute to domain‐specific weaknesses despite otherwise average cognitive ability (Kapp‐Simon et al. 2016).

In parallel, genetics has gained attention as a key contributor to variability in outcomes in children treated for NSC (Ijichi et al. 2025). Although syndromic craniosynostosis has long been associated with mutations in genes such as *FGFR2* and *TWIST1*, emerging evidence suggests that common and rare variants in genes regulating suture biology, bone development, and neural patterning may also contribute to NSC phenotypes (Timberlake et al. 2022; Topa et al. 2024). These discoveries imply that some neurodevelopmental differences may not be secondary to altered skull morphology alone, but instead reflect shared genetic influences on both cranial and brain development (Ijichi et al. 2025; Timberlake et al. 2022). This indicates that while NSC may involve complex interactions between cranial morphology, brain development, and genetic factors with possible detrimental communicative outcomes in some children, the general impact on communication abilities at age five is modest. This finding is important, as it suggests that communication difficulties observed in children with NSC should not automatically be attributed to the craniosynostosis itself. Rather, such difficulties may reflect the range of speech‐ and language difficulties expected in the general population, as well as other specific factors such as familial speech‐ and language delay/disorder, neurodevelopmental conditions, hearing history or broader developmental vulnerabilities.

Viewed from a broader clinical perspective, the results are in alignment with the CATALISE international consensus on developmental language disorder (Bishop et al. 2016, 2017). CATALISE stresses the importance of distinguishing between speech sound disorders and broader language disorders, noting that isolated phonological or articulatory difficulties typically have a favourable prognosis and should not be classified as language disorder. This distinction corresponds well with the present results, where differences were confined to speech and syntax, while global language difficulties were uncommon. Furthermore, CATALISE advocates for detailed, domain‐specific descriptions of communicative ability, an approach possible to obtain by the CCC‐2 profile analysis in this study. In addition, CATALISE highlights that developmental language disorder (DLD) frequently co‐occurs with other difficulties in domains such as attention, literacy, and behaviour, and that parental or professional concerns should warrant referral for assessment. This is consistent with Kilcoyne et al. (2021), who demonstrated that parental concern about behaviour, rather than communication per se, was the strongest predictor of referral. Finally, CATALISE underscores that language difficulties persisting at age five are likely to be enduring. The finding that the majority of children in the present cohort scored within the normative range at this age is encouraging, suggesting that while subtle vulnerabilities in speech‐ and language domains may be observed, most children with NSC do not present with broader developmental language disorder–like profiles. In a longitudinal perspective recent registry data from Sweden (Olsson et al. 2025) indicate that children with NSC generally achieve age‐appropriate academic results, with only mild and temporary difficulties in early school years. This further supports the present finding of a low prevalence of language difficulties at age five, suggesting that most children with NSC develop functional communication skills sufficient for long‐term academic success.

An interesting observation is that, when compared to published norms, scales assessing speech‐ and language aspects (syntax and speech) were rated significantly lower by guardians of children treated for NSC compared to norms. This suggests that the most noticeable difficulties in this population lie within speech‐ and language domains of communication, aligning with prior studies that reported differences in speech (articulation) and syntax (grammar) (Becker et al. 2005; Korpilahti et al. 2012; Naran et al. 2017; Shipster et al. 2003; Virtanen et al. 1999). However, when analysing the CCC‐2 on the item level, the most frequently flagged items by guardians included difficulties with correctly pronouncing /r/ or /s/, and syntactically omitting verb endings; both of which in the Swedish language are late‐acquired and may remain variably produced at around five years of age in typically developing children (Håkansson 1998; Lohmander et al. 2024; Nettelbladt and Salameh 2007). It could also be possible that guardians of children treated for NSC may be particularly attentive to their child's development, including speech and language skills, as a result of early medical involvement and ongoing clinical follow‐up. This heightened awareness may influence parental reporting on questionnaire‐based measures such as the CCC‐2.

When examining the CCC‐2 GCC outcome depending on early versus late timing of surgery, only the metopic group reached a significant result, indicating more favourable outcomes in individuals treated surgically before 6 months of age. Although speech and language outcomes have rarely been examined as a primary focus in metopic NSC, the limited available evidence suggests that communication may represent a vulnerable developmental domain in this population. Small metopic‐only studies and retrospective cohorts have reported elevated rates of speech and/or language delay, often affecting approximately one‐quarter to one‐third of children, including difficulties in expressive and receptive language and language‐based learning (Mendonca et al. 2009; Salib et al. 2026; Shimoji et al. 2015; Sidoti et al. 1996).

Several mechanisms have been proposed to explain why speech‐ and language development may be affected in metopic NSC. Premature fusion of the metopic suture restricts frontal cranial growth, changes that may persist following surgical correction (Aldridge et al. 2017; Bottero et al. 1998; Kapp‐Simon et al. 2007). Given the central role of frontal and fronto‐temporal networks in early language acquisition, executive‐language integration, and later academic language skills, disruption to these systems during sensitive developmental periods may increase vulnerability to communication difficulties. Importantly, language‐related weaknesses in NSC have frequently been observed independently of general intelligence, suggesting that global cognitive measures may underestimate functionally meaningful impairments in everyday communication (Kapp‐Simon et al. 2007) raising the possibility that studies relying primarily on global cognitive measures may overlook functionally meaningful impairments in everyday communication (Tio et al. 2026; van der Vlugt et al. 2012).

Interpretation of associations between timing of surgery and developmental outcomes is complicated by the fact that later surgical intervention may reflect underlying clinical or contextual factors rather than treatment delay alone. Children may undergo surgery at a later age due to medical comorbidities, perinatal complications, anaesthetic risk, or the need to prioritise other health concerns before craniofacial intervention. Additionally, variation in referral pathways and diagnostic timing may further influence age at surgery independently of neurodevelopmental risk. As a result, children treated later may differ systematically from those treated earlier in ways that are difficult to fully account for, underscoring the need for cautious interpretation of timing effects on developmental outcomes.

The present results contribute to a field where methodological inconsistencies have long complicated interpretation. Previous research has relied on proxies, such as therapy referral, or reported speech and/or language outcomes without clarifying whether speech and language were separately evaluated, often obtained through retrospective studies. The current study demonstrates the utility of the screening instrument CCC‐2 in providing domain‐specific insight into communicative abilities in NSC.

The use of screening rather than full standardized speech and language assessment should be considered in relation to the clinical and methodological aims of the study. The purpose was not to provide a diagnostic characterization of each child's speech and language profile, but to examine whether systematic guardian‐reported screening in an unselected cohort could identify communicative vulnerabilities at group level within routine follow‐up. In this context, the CCC‐2 offered a feasible method for collecting standardized information from a large consecutive cohort, including families who travelled long distances and children for whom lengthy direct assessment or repeated testing sessions would not be practical. The findings should therefore be interpreted as screening outcomes rather than diagnostic outcomes. Scores below the CCC‐2 cut‐off indicate children who may benefit from more detailed SLP assessment, whereas scores within the normative range do not exclude subtle difficulties that may be identified through direct testing or clinical observation.

## 5 Limitations

This study has several limitations that should be acknowledged. First, the data rely on guardian‐reported outcomes through the CCC‐2. Although this tool provides valuable insight into everyday communication, it may not fully capture subtle phonological, syntactic, or pragmatic difficulties that could be identified through direct assessment. Second, the subgroup analyses were limited by small sample sizes, particularly in the unicoronal and miscellaneous NSC groups, reducing the statistical power to detect differences across craniosynostosis types. Third, this study relies on comparison of the study group against normative data previously published and not a control group matched by the authors. Fourth is the confounder of some children possibly receiving speech‐ language therapy before 5 years of age, potentially impacting the results. Finally, the cross‐sectional design at age five prevents conclusions about developmental trajectories, making it difficult to determine whether reported speech‐ and language weaknesses persist, resolve, or evolve into literacy‐related difficulties at school age.

## 6 Clinical Implications

From a clinical perspective, these findings support the use of systematic screening to identify children who warrant more detailed evaluation. The findings of this study may not support the assumption that all children with non‐syndromic craniosynostosis require extensive speech‐ and language assessment solely on the basis of a non‐syndromic craniosynostosis diagnosis. Given the practical constraints of multidisciplinary care, screening may provide an effective way to monitor communication development across the broader patient group. Children scoring below normative ranges, children whose parents or clinicians report concerns, and children with additional developmental or medical risk factors should be referred for more comprehensive speech and language assessment. Thus, screening should be regarded as a first step in a multi‐level model rather than as a replacement for full SLP evaluation. Given that parental concern alone has been shown to be an insufficient predictor of language difficulties, structured screening tools such as the CCC‐2 remain valuable for ensuring that children treated for NSC receive referral and support when indicated. Recent qualitative research (Tio et al. 2025) also highlights that while parents often express concerns about their child's cognitive development after craniosynostosis surgery, both parents and children generally describe age‐appropriate cognitive and social functioning. Combined with the present findings showing low prevalence of communication difficulties at age five, this suggests that a majority of children treated for NSC develop communication and learning abilities within the expected range. Clear communication of these outcomes to families may help alleviate unnecessary concern.

## 7 Future Research

Future research should include direct assessment of speech, phonological processing, and syntax, as well as longitudinal follow‐up into the school years to examine possible links with literacy and academic achievement. Larger, multicentre studies would help increase statistical power for comparisons across craniosynostosis subtypes and surgical timing. In addition, integrating genetic and neuroimaging approaches could clarify the biological mechanisms underlying selective communicative vulnerabilities, thereby informing individualized monitoring and intervention strategies.

## 8 Conclusion

In conclusion, this study shows that most children treated for NSC perform within the normative range on guardian‐reported communicative abilities at age five. However, subtle weaknesses in syntax and speech were indicated by some guardians on a group level, and individual children did present with reportedly more substantial communicative issues. These findings suggest that NSC is not strongly associated with broad language disorder–like profiles but may confer selective risks in speech‐ and language aspects of communication. Screening may therefore be useful as a first step in identifying children who require more detailed evaluation. Future studies combining caregiver report with direct assessment, and extending into the school years, are needed to clarify how these early patterns relate to literacy and academic outcomes.

## Ethics Statement

The study was conducted according to the principles stated in the Declaration of Helsinki, and was approved by the Swedish Ethical Review Authority (no. 2021‐02105 and 2025‐01463‐02).

## Conflicts of Interest

The authors declare that there are no conflicts of interest. A version of the manuscript was presented at the European Society of Craniofacial Surgery (ESCFS) Congress, Helsinki, Finland, on 5–7 September 2024.

## Data Availability

Research data are not shared. The data used in this study are derived from clinical data and include sensitive personal health information. Owing to ethical restrictions, the opt‐out nature of participant inclusion, and the conditions under which the data were approved for use, the data cannot be made publicly available or shared with external parties.
